# Differential expression of the FAK family kinases in rheumatoid arthritis and osteoarthritis synovial tissues

**DOI:** 10.1186/ar2318

**Published:** 2007-10-26

**Authors:** Shiva Shahrara, Hernan P Castro-Rueda, G Kenneth Haines, Alisa E Koch

**Affiliations:** 1 Department of Medicine, Feinberg School of Medicine, Northwestern University, Chicago, Illinois 60611, USA; 2 Department of Pathology, Feinberg School of Medicine, Northwestern University, Chicago, Illinois 60611, USA; 3 Department of Pathology, Yale University School of Medicine, New Haven, Connecticut 06511, USA; 4 Veteran's Administration, Chicago Health Care System, Lakeside Division, Chicago Illinois 60611, USA; 5 Veteran's Administration and the University of Michigan Medical Center, Ann Arbor, Michigan 48109, USA

## Abstract

The focal adhesion kinase (FAK) family kinases, including FAK and proline-rich kinase 2 (Pyk)2, are the predominant mediators of integrin αvβ3 signaling events that play an important role in cell adhesion, osteoclast pathology, and angiogenesis, all processes important in rheumatoid arthritis (RA). Using immunohistochemical and western blot analysis, we studied the distribution of phospho (p)FAK, pPyk2, pSrc, pPaxillin and pPLCγ in the synovial tissue (ST) from patients with RA, osteoarthritis (OA) and normal donors (NDs) as well as in RA ST fibroblasts and peripheral blood differentiated macrophages (PB MΦs) treated with tumor necrosis factor-α (TNFα) or interleukin-1β (IL1β). RA and OA STs showed a greater percentage of pFAK on lining cells and MΦs compared with ND ST. RA ST fibroblasts expressed pFAK at baseline, which increased with TNFα or IL1β stimulation. Pyk2 and Src were phosphorylated more on RA versus OA and ND lining cells and MΦs. pPyk2 was expressed on RA ST fibrobasts but not in MΦs at baseline, however it was upregulated upon TNFα or IL1β activation in both cell types. pSrc was expressed in RA ST fibroblasts and MΦs at baseline and was further increased by TNFα or IL1β stimulation. pPaxillin and pPLCγ were upregulated in RA versus OA and ND lining cells and sublining MΦs. Activation of the FAK family signaling cascade on RA and OA lining cells may be responsible for cell adhesion and migration into the diseased STs. Therapies targeting this novel signaling pathway may be beneficial in RA.

## Introduction

In rheumatoid arthritis (RA), macrophages (MΦs) derived from circulating monocytes are key regulators of joint inflammation and destruction. Hence, suppression of cell adhesion and migration into the RA synovial tissue (ST) may ameliorate inflammation. In this study we determined integrin-associated signaling molecules that become activated, probably as a result of inflammation in RA ST. Focal adhesion kinase (FAK) and proline-rich tyrosine kinase (Pyk)2 are two members of a family of nonreceptor protein tyrosine kinases that are activated by a variety of extracellular stimuli [1]. FAK and Pyk2 associate with the cytoskeleton and with integrin-signaling complexes by binding to Src kinase and paxillin [2-5]. FAK is rapidly tyrosine phosphorylated on cell adhesion, creating a high-affinity binding site for Src and thereby increasing phospholipase C (PLC)γ enzymatic activity [6]. Paxillin is a substrate for the FAK-Src complex that functions as an adaptor molecule for various signaling and structural proteins, and can promote migration of fibroblasts, MΦs and endothelial cells [7-11].

FAK expression is ubiquitous and FAK is activated by numerous integrins, suggesting that FAK activation is common adhesion-dependent signal [12-14]. Unlike FAK, Pyk2 expression is highly cell-type and tissue specific. Pyk2 is tyrosine phosphorylated in response to stress (UV irradiation, tumor necrosis factor-α (TNFα) and hyperosmotic shock), G protein-coupled receptor agonists (angiotensin II, thrombin) and growth factors (vascular endothelial growth factor (VEGF), basic fibroblast growth factor (bFGF), and platelet derived growth factor (PDGF) [15-17]. Although FAK activation is closely tied to integrin-mediated adhesion, activation of Pyk2 can be independent of cell adhesion [18]. FAK and Pyk2 are expressed in osteoclasts, and both proteins are tyrosine phosphorylated in response to integrin αvβ3 ligation, a process which may be crucial for bone resorption [3,19]. Both FAK and Pyk2 play a central role in linking integrin αvβ3 signaling to the formation of podosomes and actin rings in osteoclasts. Although FAK is phosphorylated by Src, Pyk2 can be phosphorylated through a Src- or a Ca^2+^-dependent pathway [18]. Additionally, FAK is involved in angiopoietin-1 and VEGF-induced endothelial cell migration and angiogenesis [8,20]; however, the role of Pyk2 in endothelial cell function has not been explored.

MΦs isolated from RA ST have the potential to differentiate to osteoclasts in the presence of receptor activator of NF-kappaB ligand (RANKL) and macrophage colony stimulating factor (M-CSF) [21]. Stimulation of PB monocytes with M-CSF mediates FAK activation, suggesting that FAK may be involved in monocyte differentiation into MΦs [22]. Interestingly, in rat adjuvant induced arthritis (AIA) intra-articular injection of dominant negative FAK adenovirus reduces mononuclear cell recruitment into the joint. Inhibition of FAK suppresses VEGF-induced mononuclear cell migration into the AIA ankle [8]. This suggests that suppression of FAK activation may be important for reducing cell recruitment into RA ST.

In this study we investigated the expression pattern of pFAK, pPyk2, pSrc, pPaxillin and pPLCγ in RA and OA ST. Activation of these signaling proteins on RA and OA ST lining cells may be responsible for monocyte adhesion and migration into the diseased STs, whereas activation of these signaling proteins on MΦs may be important for both monocyte to MΦ differentiation as well as MΦ differentiation into osteoclasts.

## Materials and methods

STs were obtained from patients diagnosed with RA and OA undergoing arthroplasty or synovectomy. RA or OA were diagnosed according to the criteria of the American Collage of Rheumatology [23,24]. Normal STs, were obtained from fresh autopsies or amputations. STs, were snap frozen in OCT compound (Miles, Elkhart, Indiana, USA). All samples were obtained with Institutional Review Board approval additionally informed patient consent or consent from next of kin was documented.

### Antibodies and immunohistochemistry

STs were cut into 4 μm sections and fixed in cold acetone for 20 minutes. Endogenous peroxidase was quenched by treatment with 3% H_2_O_2 _for 5 minutes. STs were next pretreated with 3% goat sera for 1 hour at 37°C before application of primary antibody in 4°C overnight. Indirect immunoperoxidase staining was performed at 37°C for 1 hour. Polyclonal antibody (pAb) rabbit anti-human pFAK, pAb rabbit anti-human pPyk2, pAb rabbit anti-human pSrc, pAb rabbit anti-human pPaxillin and pAb rabbit anti-human pPLCγ were all purchased from Biosource (Camarillo, California, USA) or Cell Signaling Technology (Beverly, Massachusetts, USA), and were used at a concentration of 1 μg/ml. Isotype-specific IgG (rabbit) was used as a negative control. Staining was performed using Vector Elite ABC Kits (Vector, Burlingame, California, USA) and diaminobenzidine (Kirkegaard and Perry, Gaithersburg, Maryland, USA) as a chromogen.

### Microscopic analysis

Vascularity was defined as a score as follows: 1, marked decrease in vessels; 2, normal density of vessels; 3, increased density of vessels; 4, marked increase in vessel density, resembling granulation tissue. Inflammation was defined as a score as follows: 1, normal; 2, mildly increased number of inflammatory cells, arrayed as individual cells; 3, moderately increased number of inflammatory cells including distinct clusters (aggregates); 4, marked diffuse infiltrate of inflammatory cells. MΦs were distinguished from fibroblasts based on morphology and CD 11b/c immunoreactivity. Score data were pooled and the mean ± SEM was calculated in each data group [25-27]. Each of the ST components was graded for immunostaining by a frequency of attaining scale, scored 0–100% where 0% indicates no staining and 100% indicates that all cells were immunoreactive. The number of cells of a given type that reacted with a specific antibody divided by the total number of cells of that given type was defined as the percentage of reactivity. The mean percentage of reactivity was determined for 3 high power fields (HPF) in STs for each cell type and antibody analyzed. Each slide was evaluated by a single blinded pathologist (GKH). Selected sections were analyzed by an additional observer (SS).

### Cell culture and western blot analysis

RA fibroblasts were isolated from fresh STs by mincing and digesting in a solution of dispase, collagenase and DNase [28]. Cells were used at passage 4 or older, at which time they are a homogeneous population of fibroblasts. Cells were cultured in DMEM containing 10% heat-inactivated fetal bovine serum (FBS) [29]. Mononuclear cells were isolated by Histopaque (Sigma Chemical Co., St. Louis, Missouri, USA) gradient centrifugation. PB monocytes were then isolated from the mononuclear cells by Percoll (Sigma Chemical Co.) gradient centrifugation and countercurrent centrifugal elutriation (Beckman-Coulter, Fullerton, California, USA) [29]. Following adherence, monocytes were differentiated *in vitro *for 7 days in RPMI containing 20% FBS plus 1 μg/ml polymyxin B sulfate (Sigma Chemical Co). As PB monocytes were isolated from buffy coats by elutriation polymyxin B was added to the media preventatively. The endotoxin levels in RPMI, FBS and PBS as measured by Limulus Amebocyte Lysate (LAL) (QCL-1000; Cambrex Bioscience, Maryland, USA) were below the lowest detectable level of 0.1 endotoxin unit (EU). The endotoxin levels in TNFα and IL1β were lower than 1.0 EU per 1 μg of the cytokine as determined by the LAL method.

RA ST fibroblasts (cultured in DMEM with 10% FBS) and MΦs (cultured in RPMI with 20% FBS) were either untreated or treated with TNFα (10 ng/ml; R&D Systems, Minneapolis, New Mexico, USA) [30] or IL1β (10 ng/ml; R&D Systems) [31] for 0 to 120 min.

Western blot analysis was conducted as previously described [29]. Briefly, 60 μg of each sample was loaded on a 10% SDS-PAGE gel and transferred to nitrocellulose membranes using a semi-dry transblotting apparatus (Bio-Rad, Hercules, California, USA). Nitrocellulose membranes were blocked with 5% nonfat milk in Tris-buffered saline Tween (TBST) buffer-20 mM Tris, 137 mM NaCl, pH 7.6, with 0.1% Tween for 60 min at room temperature. Blots were probed with rabbit anti-pFAK (Tyr 576/577), anti-pPyk2 (Tyr 402), or anti-pSrc (Tyr 527) (Cell Signaling Technology) overnight and after stripping reprobed with rabbit anti-FAK, anti-Pyk2 or anti-Src (Cell Signaling Technology at 1:1000) overnight.

### Statistical analysis

The data was analyzed using Student's *t*-tests. *P *values less than 0.05 were considered significant.

## Results

### pFAK localization

**Figure 1:**
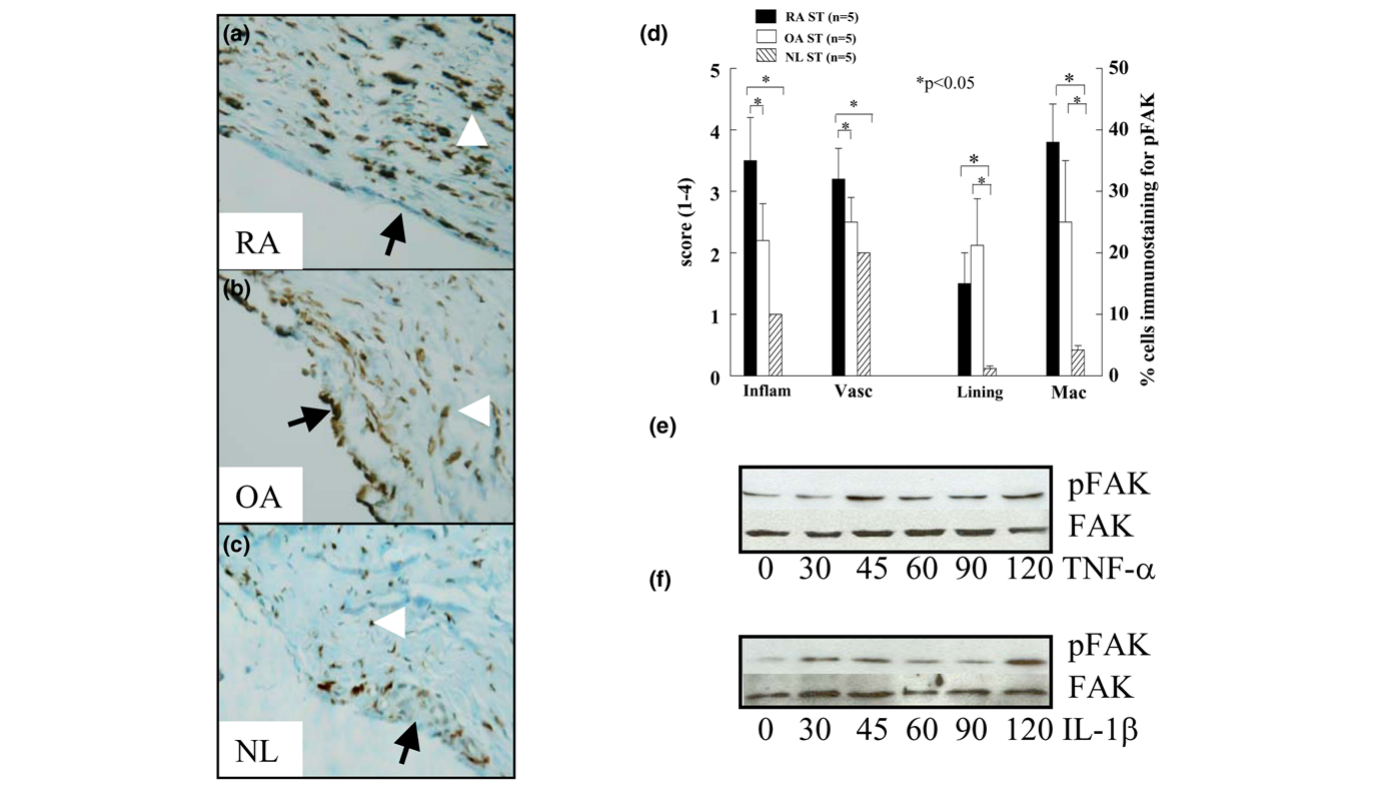
pFAK expression pattern in RA, OA and normal donor (ND) ST. **(a)** Rheumatoid arthritis synovial tissue (RA ST) stained with anti-pFAK, showing positive staining of the lining cell layer (black arrow) and subsynovial MΦs (white arrowhead) (×200). **(b)** Positive staining in osteoarthritis (OA) ST (×200). **(c)** Low pFAK reactivity in normal ST lining (arrow) and subsynovial macrophages (arrowhead). **(d)** The quantification of data obtained from a, b and c. Bars represent the mean and SEM. Inflam, inflammatory score; Vasc, vascularity score; Lining, ST lining cell layer; Mac, subsynovial MΦs. *P < 0.05. n, numbers of patients. RA ST fibroblasts were stimulated with **(e) **tumor necrosis factor-α (TNF-α) (10 ng/ml) or **(f)** interleukin-1β(IL-1β) (10 ng/ml) from 0–120 min. Cell lysates were examined by western blot analysis for pFAK or FAK expression. The results are representative of three experiments.

As expected, the inflammatory and vascularity scores were higher in RA ST in comparison to OA and NDs. pFAK, was expressed on ST lining cells in RA patients (mean of 15% positive cells) and OA (21%) more than on ND ST lining (1%) (*P *=< 0.05) (Figure 1). pFAK staining on MΦs was also significantly higher in RA (39%) and OA (25%) compared to ND (4%) (*P *< 0.05). A few RA patients had positive immunostaining for pFAK on ST endothelial cells and lymphocytes. Unstimulated RA ST fibroblasts expressed pFAK; however, the expression increased with TNFα stimulation at 45 min and stayed upregulated until 120 min (Figure 1e). Similarly, IL1β increased pFAK expression at 30, 45 and 120 min in RA ST fibroblasts (Figure 1f). pFAK was not detected in MΦs with or without TNFα or IL1β stimulation.

### pPyk2 expression pattern

**Figure 2:**
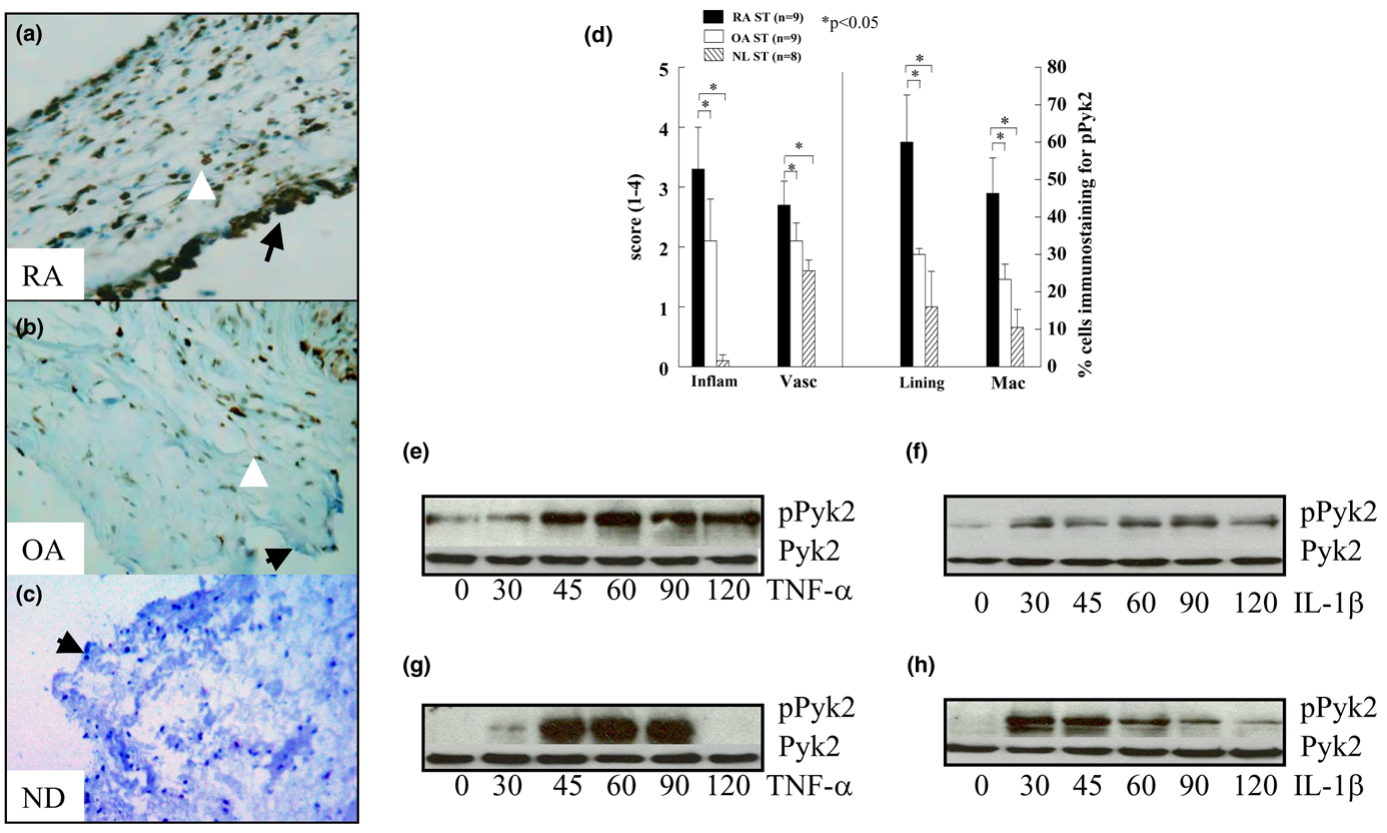
Rheumatoid arthritis synovial tissue (RA ST) had higher pPyk2 immunopositive cells compared to osteoarthritis (OA) ST. **(a)** RA (×200), compared to **(b)** OA (×200) and **(c)** normal donor (ND) (×200). **(d)** is the quantification data obtained from figure a and b. Bars represent mean and SEM.  RA ST fibroblasts **(e and f)** or peripheral blood differentiated MΦs **(g and h)** were stimulated with TNF-α (10 ng/ml) or IL1-β (10 ng/ml) from 0–120 min. Cell lysates were examined by western blot analysis for pPyk2 or Pyk2 expression. The results are representative of three experiments. Inflam, inflammatory score; Vasc, vascularity score; Lining, ST lining cell layer; Mac, subsynovial MΦs.

pPyk2 is one of the members of the nonreceptor protein tyrosine kinase FAK family and shares approximately 45% sequence homology with FAK. Both proteins are important for integrin-mediated adhesion and osteoclastogenesis [18]. pPyk2 immunostaining on ST lining and MΦs was significantly higher in RA (lining cells = 60% and MΦs = 46%) compared to OA (lining cells = 30% and MΦs = 23%) and ND (lining cells= 17% and MΦs = 10%) (*P *< 0.05) (Figure 2). However, no difference was detected in pPyk2 lining cells and MΦ immunostaining in OA and ND STs. The relative pattern of pFAK and pPyk2 expression was similar in RA patient MΦs (pFAK = 39%, pPyk2 = 46%); however, pPyk2 was highly expressed on synovial lining (pFAK = 15%, pPyk2 = 60%) compared to pFAK. The relative pattern of pFAK and pPyk2 expression was similar in OA patients' synovial lining (pFAK = 21%, pPyk2 = 30%) and MΦs (pFAK = 25%, pPyk2 = 23%). Interestingly, although pFAK was similarly expressed in RA and OA patients, the percentage of pPyk2 positive cells was significantly higher in RA ST lining and sublining compared to that of OA and ND. Rarely, RA patients showed positive immunostaining for pPyk2 on ST endothelial cells, fibroblasts and lymphocytes. pPyk2 was detected on unstimulated ST fibroblasts, and the expression was further increased by TNFα and IL1β stimulation and stayed upregulated up to 120 min (Figure 2d,e). MΦs did not express pPyk2 at baseline; however, after 30 to 45 min stimulation with TNFα or IL1β a robust level of pPyk2 was detected. In MΦs, pPyk2 remained activated for 90 min subsequent to TNFα or IL1β activation and thereafter markedly decreased (Figure 2f, h).

### pSrc localization

**Figure 3:**
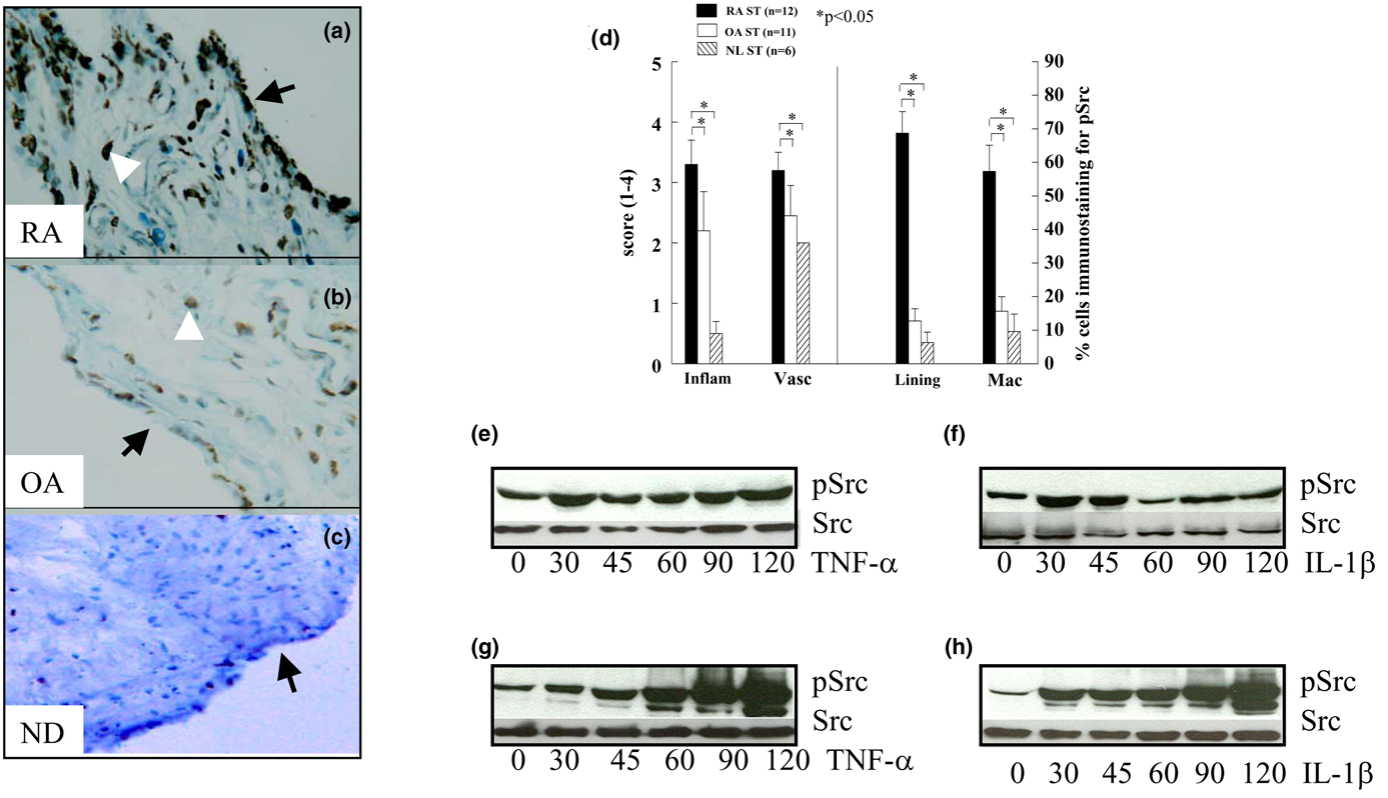
pSrc is upregulated in rheumatoid arthritis synovial tissue (RA ST) compared to osteoarthritis (OA) ST. **(a)** RA (×200), compared to **(b)** OA (×200) and **(c)** normal donor (ND) (×200). **(d)** is the quantification data obtained from a and b. Bars represent mean and SEM. RA ST fibroblasts **(e and f)** or peripheral blood differentiated MΦs **(g and h)** were stimulated with TNF-α (10 ng/ml) or IL-1β (10 ng/ml) from 0–120 min. Cell lysates were examined by western blot analysis for pSrc or Src expression. The results are representative of three experiments. Inflam, inflammatory score; Vasc, vascularity score; Lining, ST lining cell layer; Mac, subsynovial MΦs.

Integrin αvβ3 activation induces FAK and Pyk2 phosphorylation via Src. Phosphorylation of FAK and Pyk2 results in formation of a signaling complex consisting of signaling molecules including Src and paxillin. Formation of a Pyk2-Src complex and the kinase activity of Src is required for bone resorption by osteoclasts [32]. Src is highly phosphorylated in RA ST lining cells (RA = 68%) and MΦs (RA = 57%) compared to OA (lining cells = 13%, MΦs = 16%) and ND ST (lining cells = 6%, MΦs = 10%) (Figure 3). Although pSrc associated with both FAK and Pyk2 signaling complexes, the expression pattern of pSrc in RA ST lining (pSrc = 68%, pPyk2 = 60%) and sublining (pSrc = 57%, pPyk2 = 46%) was similar to pPyk2, while pSrc immunostaining in OA ST lining (pSrc = 12%, pPyk2 = 30%, pFAK = 21%) and sublining (pSrc = 15%, pPyk2 = 23%, pFAK = 25%) was comparable to both Pyk2 and FAK. RA ST endothelial cells and lymphocytes were occasionally immunopositive for pSrc. Both RA ST fibroblasts and differentiated MΦs expressed pSrc at baseline. pSrc remained activated in RA ST fibroblasts stimulated with TNFα up to 120 min (Figure 3d). In contrast, IL1β induced activation of pSrc no longer than 45 min in RA ST fibroblasts (Figure 3e). In MΦs, both TNFα and IL1β mediated robust activation of pSrc in a time dependent manner up to 120 min (Figure 3f and 3h).

### pPaxillin immunostaining

**Figure 4:**
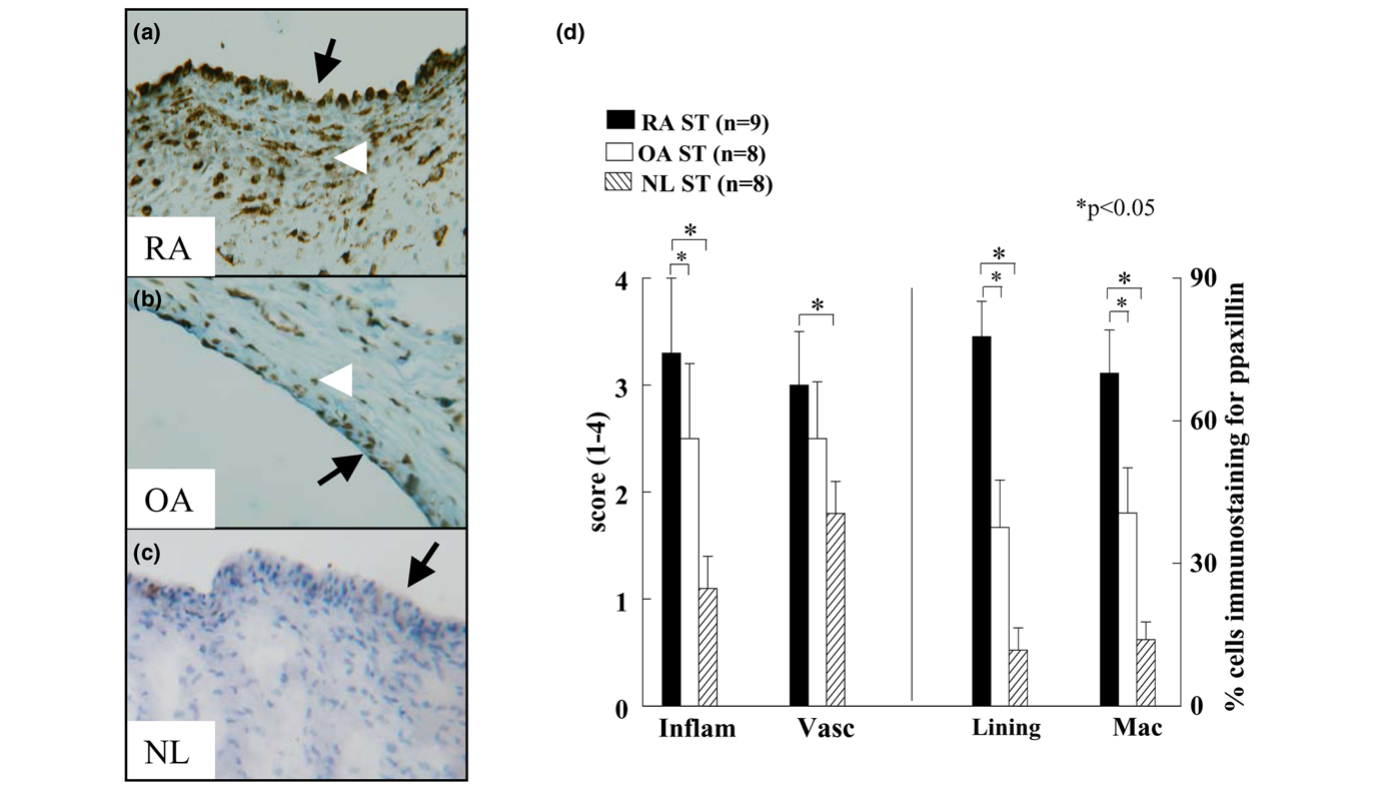
Immunostaining of pPaxillin is increased in rheumatoid arthritis synovial tissue (RA ST) compared to osteoarthritis (OA) and normal donor (ND) ST. (a) demonstrates RA ST stained with anti-pPaxillin (×200), (b) shows positive staining in OA ST (×200). (c) Low pPaxillin reactivity in normal ST lining and subsynovial MΦs. (d) Is the quantification data obtained from a, b and c. Bars represent mean and SEM. Inflam, inflammatory score; Vasc, vascularity score; Lining, ST lining cell layer; Mac, subsynovial MΦs.

Paxillin is a multidomain adaptor protein that interacts with signaling proteins such as FAK, Pyk2, Src and PLCγ [6,33]. The phosphorylation of paxillin is modulated by cell adhesion. Paxillin is recruited to the FAK and Pyk2 signaling complex upon integrin αvβ3 ligation in osteoclasts. We found that pPaxillin is expressed on ST lining cells in RA patients (77%) to a significantly higher degree than OA (37%) and ND (12%) (*P *< 0.05). pPaxillin immunostaining on MΦs was also significantly higher in RA (70%) compared to OA (40%) and ND (14%) (*P *< 0.05) (Figure 4). Similar to pSrc and pPyk2, paxillin was highly phosphorylated in RA ST lining (pPaxillin = 77%, pSrc = 68%, pPyk2 = 60%) and sublining (pPaxillin = 70%, pSrc = 57%, pPyk2 = 46%). In contrast, pPaxillin immunostaining on OA lining (pPaxillin = 37%, pPyk2 = 30%, pFAK = 21%) and sublining (pPaxillin = 40%, pPyk2 = 23%, pFAK = 25%) was comparable to pFAK and pPyk2. Our findings suggest that the colocalization and activation of FAK, Pyk2, Src and paxillin in RA and OA patient's ST lining and sublining may be important for integrin-mediated signaling.

### pPLCγ expression pattern

**Figure 5:**
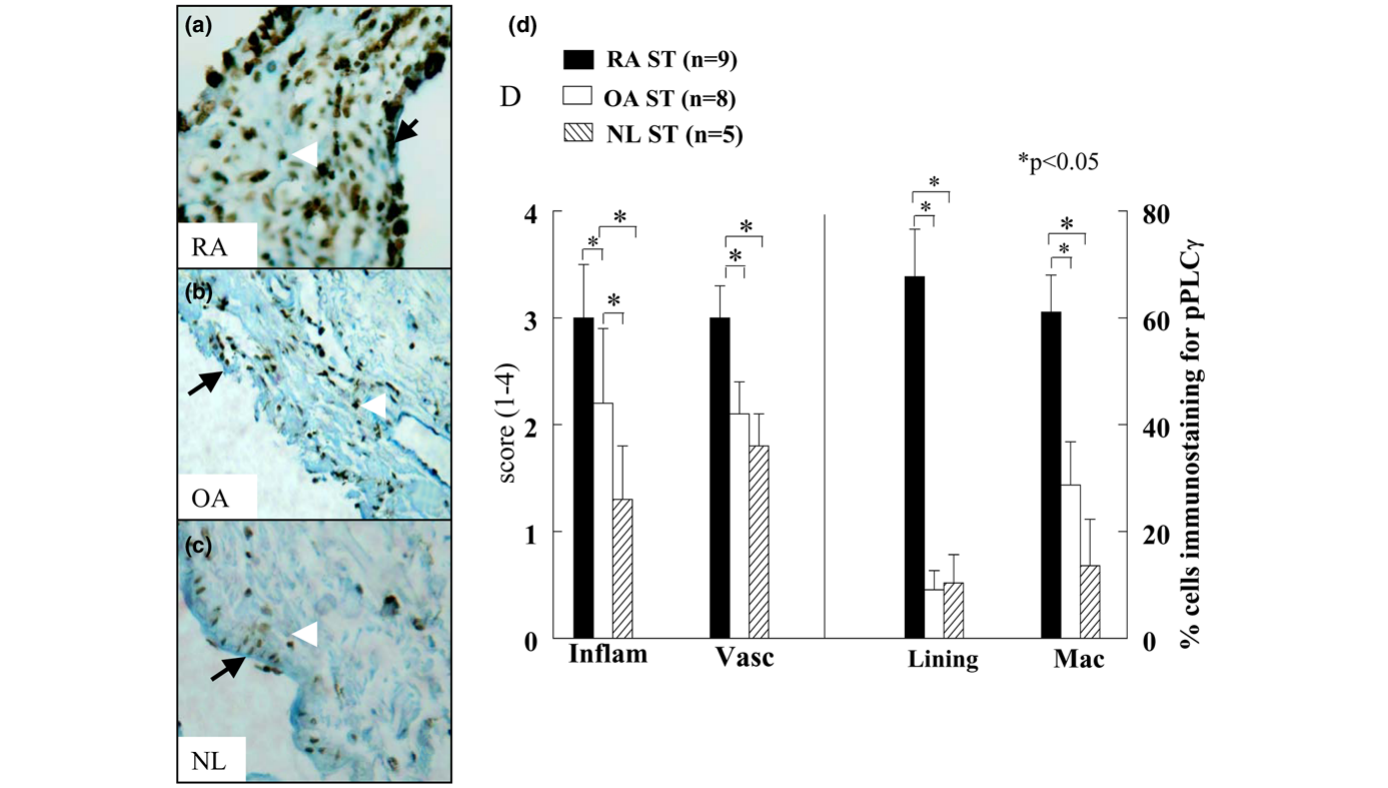
pPLCγ immunostaining is higher in rheumatoid arthritis synovial tissue (RA ST) in comparison to osteoarthritis (OA) and normal donor (ND) ST. **(a)** RA (×200), compared to **(b)** OA (×200) and **(c)** ND (×200). **(d)** The quantification data obtained from a, b and c. Bars represent mean and SEM. Inflam, inflammatory score; Vasc, vascularity score; Lining, ST lining cell layer; Mac, subsynovial MΦs.

Upon αvβ3 integrin-mediated adhesion, PLCγ associates with the Pyk2 and FAK signaling complex [6,33]. M-CSF can also induce association of αvβ3 integrins with PLCγ, PI3K and Pyk2 in a Src-independent manner [33]. The inflammatory and vascularity scores for pPLCγ immunostaining were higher in RA ST in comparison to OA and ND. RA patients most strongly expressed pPLCγ in the lining (67%) and on MΦs (61%) in ST, compared to OA patients (lining = 9%, MΦs = 28%) and ND subjects (lining = 10%, MΦs = 14%)(Figure 5). Interestingly pPLCγ immunostaining was similar on OA and ND MΦs. The positive immunostaining of pPLCγ was comparable to pPyk2 expression on RA ST lining (pPLCγ = 67%, pPyk2 = 60%) and sublining (pPLCγ = 61%, pPyk2 = 46%). Whereas, pPLCγ immunostaining on OA lining (pPLCγ = 9%, pPyk2 = 30%) was lower than that of pPyk2. These results suggest that Pyk2 and its associated signaling protein complex, namely Src, paxillin, and PLCγ are activated on the RA ST lining and MΦs to a greater extent than on OA ST. A few RA patients had positive immunostaining for pPLCγ on fibroblasts and lymphocytes.

## Discussion

**Figure 6:**
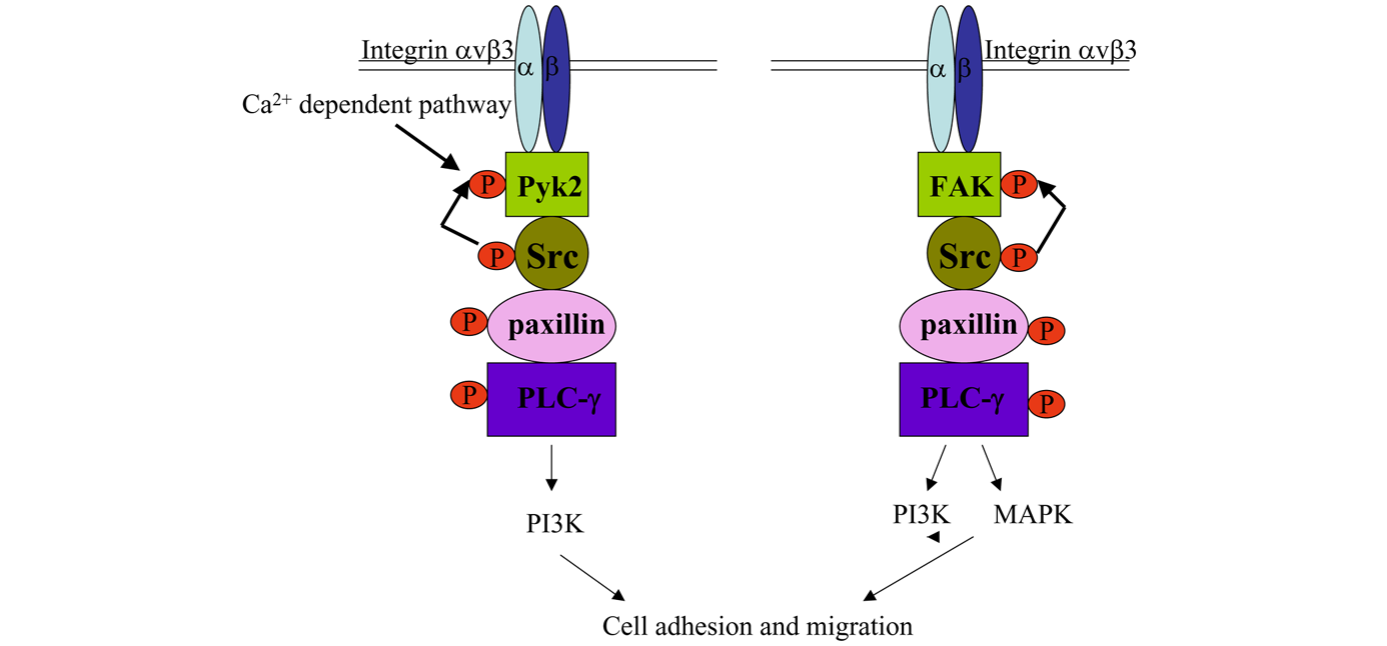
Putative integrin signaling pathways through Pyk2 or FAK. In response to integrin αvβ3 activation, Pyk2 and/or FAK are recruited to a signaling complex that consists of Src, paxillin and PLCγ. Pyk2 may be phosphorylated through Src or other Ca^2+ ^dependent pathways whereas FAK is phosphorylated through Src. Both Pyk2 and FAK can result in activation of PI3K and/or MAPK that may lead to cell adhesion and migration into the rheumatoid arthritis synovial tissue (RA ST).

RA is a chronic inflammatory disease characterized by synovial hyperplasia. Proliferation of synovial cells leads to pannus formation resulting in progressive bone and joint destruction. It has been reported that FAK and Pyk2 are involved in integrin αvβ3-mediated bone resorption [19,34-36]. Interestingly little is known about the activation of these proteins in RA ST. In this study we demonstrated the phosophorylation of FAK and Pyk2 as well as their downstream signaling molecules, namely, Src, paxillin and PLCγ in arthritic ST (Figure 6). In addition, we determined differences between diseased and ND STs in regards to these molecules.

Angiogenesis is important in the growth and proliferation of the RA ST pannus. FAK exerts its pro-angiogenic effects through multiple mechanisms. Angiopoietin-1 induced endothelial cell sprouting through FAK [37]. VEGF-mediated endothelial cell migration and tube formation occurred through FAK phosphorylation and subsequent PI3K activation [7]. Additionally, VEGF induced FAK tyrosine phosphorylation in RAW cells.

VEGF-mediated RAW cell chemotaxis was suppressed by dominant-negative FAK adenovirus [8]. In rat AIA, intra-articular injection of dominant negative adenoviral FAK reduced mononuclear cell recruitment into the joint [8]. Inhibition of FAK phosphorylation suppressed 3T3 fibroblast and human umbilical vein endothelial cell migration [9]. These results suggest that activation of the FAK signaling pathway may be important for fibroblast, macrophage and endothelial cell migration, all processes involved in RA ST inflammation and proliferation.

Upon localization to the integrin cluster, FAK becomes autophosphorylated and activates a number of downstream targets such as Src and PI3K. Src recruitment results in phosphorylation of several other residues associated with FAK, including paxillin [38]. Overexpression of FAK and PLCγ in COS-7 cells increases PLCγ enzymatic activity and tyrosine phosphorylation. FAK-induced PLCγ phosphorylation may be due to FAK interaction and activation of Src family kinases [6]. Our results demonstrate that unlike Src, paxillin and PLCγ, which are highly phosphorylated in RA ST lining, only low numbers of RA ST lining cells are immunopositive for pFAK. pFAK staining in RA ST sublining was not greater than that in OA ST. Nevertheless, pFAK staining in RA ST lining and sublining was significantly higher than in ND STs. We additionally showed that FAK, Pyk2 and Src were phosphorylated on unstimulated RA ST fibroblasts. The pro-inflammatory cytokines TNFα and IL1β further activated pFAK, pPyk2 and pSrc expression up to 120 min. The activation of the FAK-Src-paxillin-PLCγ pathway in RA ST lining and sublining suggests that these signaling proteins may be important for cell adhesion, cell migration and perhaps even MΦ differentiation to osteoclasts.

MΦs isolated from Pyk2-null mice showed impaired migration in response to chemokine stimulation. Ca^2+ ^release in response to chemokine stimulation as well as integrin-mediated activation of PI3K were compromised in Pyk2-/- MΦs [39]. These findings suggest that Pyk2 plays an important role in MΦ function modulating migration [39]. Integrin αvβ3-mediated signaling is dependent on the phosphorylation of Pyk2, Src, paxillin and PLCγ. However, M-CSF can induce osteoclast differentiation by recruiting Pyk2, PLCγ and PI3K in a Src-independent manner, an effect which is blocked by PLC inhibitors [33]. These finding suggest that in the absence of one Pyk2 family member, other signaling proteins can rescue osteoclast function. Previous findings indicate that similar to FAK, Pyk2 is essential for integrin-mediated adhesion, monocyte-MΦ migration, and osteoclast signaling and differentiation [18]. Our data demonstates that pPyk2, pSrc, pPaxillin and pPLCγ are similarly expressed on RA ST lining and sublining, and this expression is significantly higher than that found in OA and ND ST. The RA ST lining layer consists of fibroblasts and MΦs that are in close proximity to one another. Although, pPyk2 is undetected on MΦs or expressed in low levels on RA ST fibroblasts, the presence of TNFα and IL1β retains pPyk2 activation both in MΦs and RA ST fibroblasts.

In RA ST fibroblasts, baseline levels of pSrc are greater than that of pFAK and pPyk2. Furthermore, TNFα and IL1β treatment increased pSrc expression modestly in RA ST fibroblasts. However, both cytokines induced pSrc levels to a greater extent in MΦs. Src, paxillin and PLCγ are associated with both integrin activation of FAK and Pyk2 pathways. It is controversial whether Src kinase activity is essential for Pyk2-induced osteoclast bone resorption as Pyk2 can be phosphorylated in a Ca^2+^-dependent manner [40]. In contrast, activation of Src is necessary for FAK function.

Circulating leukocytes, including lymphocytes and MΦs, express high levels of paxillin [41]. Paxillin is a multi-domain adapter molecule which is important for recruiting multiple signaling protein to a specific location within the cell [42]. Paxillin is modulated in response to integrin-mediated cell adhesion and growth factors. In this study we demonstrate that pPaxillin is detected twofold higher in RA ST lining and sublining compared to OA ST. The numbers of pPaxillin immunopositive cells are significantly lower in ND ST lining and sublining compared to RA ST, suggesting that activation of paxillin is associated with RA inflammation.

PLCγ-null fibroblasts are defective in adhesion, spreading and migration [43]. Integrin engagement by fibronectin induces tyrosine phosphorylation of PLCγ1 and that this signaling event requires Src activity [43]. Further, paxillin associates with PLCγ1 in cells grown on fibronectin that may be mediated by FAK or Pyk2 [44]. Although pPLCγ is significantly upregulated on RA ST lining and sublining, it is similarly expressed in OA and ND ST.

Multiple signal-transduction pathways have been implicated in RA, most notably protein kinases such as MAPK [45] and PI3K [46]. Preclinical models have confirmed the therapeutic potential of p38 MAPK [47,48] and PI3K [49] inhibition and clinical trials are under way to evaluate inhibitors for these signaling pathways [45]. Although the preliminary results obtained from animal models are promising, proof of safety has not yet been obtained. As both p38 MAPK and PI3K are involved in normal processes, inhibition of these signaling pathways may produce untoward effects. Hence, identifying the intermediary signaling proteins that are dysfunctional in RA and not in ND may offer new therapeutic options.

## Conclusion

Taken together, our results demonstrate that FAK family kinases, including FAK and Pyk2, and their associated signaling intermediates, namely Src, paxillin and PLCγ are phosphorylated in RA ST lining and sublining. Although both FAK and Pyk2 have been implicated in cell adhesion, migration and osteoclast differentiation, alternative pathways may be used for each function. Inhibiting activation of the FAK superfamily may suppress cell adhesion and migration into RA ST and provide a novel therapeutic target.

## List of abbreviations

AIA = adjuvant induced arthritis; FAK = focal adhesion kinase; FGF = fibroblast growth factor; IL = interleukin; M-CSF = macrophage colony stimulating factor; ND = normal donor; OA = osteoarthritis; PB MΦ = peripheral blood differentiated macrophages; PDGF = platelet derived growth factor; PLC = phospholipase C; Pyk = proline rich kinase; RA = rheumatoid arthritis; RANKL = receptor activator of NF-kappa B ligand; ST = synovial tissue; TBS-T = Tris-buffered saline Tween; TNF = tumor necrosis factor; VEGF = vascular endothelial growth factor.

## Competing interests

The authors declare that they have no competing interests.

## Authors' contributions

SS was responsible for design of the study, acquisition of data, analysis and interpretation of the data and manuscript preparation; HPR and GKH were responsible for acquisition of data; AEK was responsible for design of the study, interpretation of the data and manuscript preparation, and all authors have approved the content of the manuscript.
